# Effective mitigation of the belief perseverance bias after the retraction of misinformation: Awareness training and counter-speech

**DOI:** 10.1371/journal.pone.0282202

**Published:** 2023-03-08

**Authors:** Jana Siebert, Johannes Ulrich Siebert

**Affiliations:** 1 Department of Applied Economics, Faculty of Arts, Palacky University Olomouc, Olomouc, Czech Republic; 2 Department of Business and Management, Management Center Innsbruck, Innsbruck, Austria; University of Massachusetts Lowell, UNITED STATES

## Abstract

The spread and influence of misinformation have become a matter of concern in society as misinformation can negatively impact individuals’ beliefs, opinions and, consequently, decisions. Research has shown that individuals persevere in their biased beliefs and opinions even after the retraction of misinformation. This phenomenon is known as the belief perseverance bias. However, research on mitigating the belief perseverance bias after the retraction of misinformation has been limited. Only a few debiasing techniques with limited practical applicability have been proposed, and research on comparing various techniques in terms of their effectiveness has been scarce. This paper contributes to research on mitigating the belief perseverance bias after the retraction of misinformation by proposing counter-speech and awareness-training techniques and comparing them in terms of effectiveness to the existing counter-explanation technique in an experiment with N = 251 participants. To determine changes in opinions, the extent of the belief perseverance bias and the effectiveness of the debiasing techniques in mitigating the belief perseverance bias, we measure participants’ opinions four times in the experiment by using Likert items and phi-coefficient measures. The effectiveness of the debiasing techniques is assessed by measuring the difference between the baseline opinions before exposure to misinformation and the opinions after exposure to a debiasing technique. Further, we discuss the efforts of the providers and recipients of debiasing and the practical applicability of the debiasing techniques. The CS technique, with a very large effect size, is the most effective among the three techniques. The CE and AT techniques, with medium effect sizes, are close to being equivalent in terms of their effectiveness. The CS and AT techniques are associated with less cognitive and time effort of the recipients of debiasing than the CE technique, while the AT and CE techniques require less effort from the providers of debiasing than the CS technique.

## Introduction

Decision making shapes important outcomes for individuals, organisations and societies [1]. If the decisions are based on misinformation, they are likely to be suboptimal. Misinformation, thus, can have serious consequences for individuals and society. Salient examples are the decisions related to the COVID-19 pandemic [2], climate change [3], Brexit [4] and the 2016 US presidential election [5]. Misinformation has always been a part of our society. However, the internet and the rise of social media platforms have facilitated its spread. According to the Eurobarometer on fake news and online disinformation [6], 37% of the respondents come across fake news every or almost every day, and 83% of the respondents believe that fake news represents a danger to democracy.

This paper uses the term ‘misinformation’ to refer to information that is initially presented as accurate but later appears to be false, regardless of any intent to mislead. Thus, misinformation in this context covers everything from timely news coverage of unfolding events requiring occasional corrections of earlier statements (with no intention to mislead the news consumers) over fake news (intentionally designed to mislead the news consumers) to retracted research papers (for reasons ranging from concerns over the quality of the data to fabrication).

Research has shown that simple retraction of misinformation is insufficient to eliminate its influence. On the one hand, individuals may persevere in opinions and judgements even after the initial information on which the beliefs or opinions were based has been retracted; this phenomenon is known as the belief perseverance bias (BPB) [7]. On the other hand, retracted misinformation may continue to influence our inferential reasoning; this phenomenon is known as the continued influence effect (CIE) [8].

The extant research on reducing the negative impact of misinformation has mainly focused on the CIE and its mitigation [8–15]. Conversely, the research on BPB is relatively limited and has focused primarily on demonstrating the bias in an experimental setting and studying underlying mechanisms [16–21]. Although several techniques to mitigate BPB have been introduced and their effectiveness tested in experiments [22–24], research comparing various debiasing techniques in terms of their effectiveness in mitigating BPB is scarce. To the best of our knowledge, the only limited comparison of two BPB debiasing techniques has been conducted by Anderson [22]. Moreover, the practical applicability of the existing BPB debiasing techniques is limited. This paper contributes to the research on mitigating the BPB by proposing two new debiasing techniques with higher practical applicability and comparing them in an experiment in terms of their effectiveness.

### Belief perseverance bias

BPB is defined in the Encyclopedia of Social Psychology as ‘the tendency to cling to one’s initial belief even after receiving new information that contradicts or disconfirms the basis of that belief’ [7, p.109]. An essential determinant of BPB is causal thinking [18]. Individuals spontaneously create causal explanations for an observed event or a particular claim. These causal explanations become accessible in memory and independent of the original claim or the observed event on which they were based. When the original claim or the basis of the observed event is later retracted, causal explanations remain available and sustain the biased belief. Numerous studies have supported the relevance of causal thinking for inducing BPB. It has been shown that inducing subjects to write causal explanations increases BPB [16], and inducing subjects to write causal explanations for an alternative claim mitigates BPB [17, 22–24]. Moreover, the availability of causal arguments supporting a belief has been shown to be positively correlated with BPB [25].

BPB is closely related to confirmation bias [19, 26], which refers to the tendency to search for and interpret evidence in ways supporting an existing belief or opinion, thus influencing reasoning and decision making [19]. After people have formed an initial opinion, BPB makes this opinion persevere, and cognitive dissonance arises in response to information contradicting their opinion. Confirmation bias helps these individuals reduce cognitive dissonance and maintain BPB by paying more attention to confirming rather than contradicting information.

Research on BPB has traditionally used a paradigm in which participants receive information on a specific topic, which is later retracted [16]. When participants are later asked for their opinion on the topic, BPB makes the biased opinion persevere, and participants do not return to their initial opinion. This paper also uses this paradigm. The BPB paradigm differs significantly from the paradigm traditionally used in CIE-related research on misinformation. In the traditional CIE paradigm [10], participants receive several small pieces of information about a particular unfolding event, one of which is later retracted. When participants are later asked specific open-ended inference questions about the event, CIE makes them refer to the retracted piece of information. Nevertheless, it has to be noted that the current research on CIE is moving away from this traditional paradigm by not limiting itself to unfolding events and providing information in small pieces or measuring the CIE of misinformation by using open-ended inference questions related to misinformation [14, 27, 28].

### Techniques to reduce the negative impact of misinformation

Various techniques have been explored to reduce the negative impact of misinformation on individuals. These techniques can be grouped into (a) prebunking interventions and (b) debunking interventions.

#### (a) Prebunking interventions

are applied before encountering misinformation to prevent or reduce later reliance on the misinformation. Among the prebunking interventions, up-front warnings, inoculations and literacy training interventions have received particular attention.

**Up-front warnings** consist of warning people that the information to be presented might be misleading. Research has shown that up-front warnings can reduce but not eliminate later reliance on misinformation [9, 29, 30]. Ecker et al. [9] showed that specific up-front warnings explaining the CIE are more effective than general warnings (such as that the information is sometimes not double-checked before the release) in reducing the continued influence of information after retraction.

**Inoculation,** based on the inoculation theory of McGuire [31], involves, besides up-front warnings, also exposing people to particular examples of how they may be misled. Inoculation research focused initially on inoculation against specific arguments and misleading techniques on a particular topic of misinformation [32, 33]. However, due to the speed at which misinformation is currently produced, this approach is not feasible [34]. Thus, recent research has focused on inoculating the public against the manipulation techniques used to misinform in general instead of designing inoculation for the specific information content [34]. For example, Cook, Lewandowsky and Ecker [35] applied inoculations explaining the misleading effect of false-balance media coverage and the ‘fake-experts’ technique against misinformation related to climate change. Tay et al. [14] applied inoculation against the ‘fake-experts’ and anecdotes manipulation techniques and studied the effectiveness of such inoculation applied before (i.e. prebunking) and after (i.e. debunking) presenting misinformation. They found that the debunking intervention was more effective than the prebunking intervention. Lewandowsky and Yesilada [36] applied inoculation against radical-Islamist and Islamophobic disinformation using a video explaining common misleading rhetorical techniques. Roozenbeek and van der Linden [37] introduced the online fake news inoculation game *Bad News*, in which the players learn through play about techniques commonly used to produce misinformation. Buczel et al. [15] applied inoculation consisting of an adapted version of the specific warning proposed by Ecker et al. [9] and refutational preemption. The refutational preemption consisted of 1) presenting a short scenario involving misinformation and its correction, 2) asking the participants for their belief in misinformation and giving them short feedback depending on their answer, and 3) presenting other examples of misinformation, their debunking and the mechanism of reliance on misinformation.

**Literacy training interventions**, such as information, media and news literacy training, can contribute to more thoughtful news consumption [38]. Recently studied information, media, and news literacy training interventions commonly include warnings about misinformation and provide tools to identify it. Thus, they are sometimes categorised as inoculation techniques [39].

One effective way to foster information, media, and news literacy skills is through games. Wilson et al. [40] designed two educational games to teach information literacy skills to students and showed that both games transfer the targeted skills. Grace and Hone [41] developed a computer game to improve news literacy and combat fake news. Chang et al. [42] developed a news literacy card game to teach strategies for identifying misinformation and showed that students playing the game could apply the news literacy strategies to real-life contexts.

Another recently examined method to foster information, media and, in particular, news literacy is through news literacy messages. However, the research on the effectiveness of news literacy messages has shown mixed results. Tully, Vraga and Bode [43] examined the effect of two types of news literacy tweets accompanied by a picture on the perception of misinformation; one tweet highlighted that personal views influence news choices and interpretation, and the other tweet provided tips for recognising fake news. Only the second type of news literacy tweets effectively decreased the perceived credibility of misinformation. Vraga, Bode and Tully [44] later combined the same two types of tweets with expert corrections (short-format refutations belonging to debunking interventions discussed later in this section) and found that the news literacy tweets did not enhance the effectiveness of expert corrections. Hameleers [45] examined the effect of a news media literacy message consisting of three components: a) warning about the existence of misinformation; b) informing how misinformation may be detected by looking at the source and type of evidence; c) distinguishing the external reality from the biased media depiction of reality. He showed that such a message lowers the perceived accuracy of misinformation and that combining the news media literacy message with a detailed refutation is more effective in lowering the issue agreement than the news media literacy message or the detailed refutation alone. Vraga et al. [39] examined the effects of a brief news literacy video on the perception of video misinformation and found that the news literacy video did not create resistance to misinformation. These mixed results suggest that more research is needed to create effective (news) literacy messages.

#### (b) Debunking interventions

are applied after the misinformation has been presented to reduce reliance on misinformation retroactively. Typical examples of debunking interventions are refutations (also called corrections) and post-warnings.

**Refutations** have received particular attention in debunking interventions. In contrast to retractions, which simply state that the original information is incorrect, refutations provide an alternative explanation for why the original information was incorrect. It is recommended to use alternative explanations with the same explanatory relevance and complexity as the original misinformation [9, 46].

The effectiveness of both short-format (one-sentence) refutations [44, 47] and long-format (containing a greater level of explanatory details) refutations [28, 48] has been investigated. Numerous studies have shown that refutations reduce the negative impact of misinformation [49] and are more effective than simple retractions [50]. Moreover, refutations appear to be more effective than up-front warnings [49], and long-format refutations appear to be more effective than short-format refutations [28].

**Post-warnings** warn retroactively about the presence of misinformation. To the best of our knowledge, post-warnings have not been studied in BPB and CIE paradigms. However, numerous studies have examined the effectiveness of post-warnings in the eyewitness misinformation paradigm [51]. In this paradigm, an individual is an eyewitness of an event (step 1), receives a post-event narrative including misinformation (step 2) and finally is questioned about the event (step 3) [52]. A misinformation effect occurs when the individual refers to the misinformation content rather than the event’s content. Post-warnings are applied in this paradigm after receiving post-event misinformation. They include a warning about misinformation in the post-event narrative and often discredit the post-event-narrative source [51]. Numerous studies have shown the effectiveness of post-warnings in reducing the negative impact of misinformation [51, 52].

None of the studies on reducing the negative impact of misinformation reviewed above examined the effectiveness of prebunking and debunking interventions in the BPB paradigm, and only a few studies examined the effectiveness in the CIE paradigm [9, 14]. Indeed, the majority of the studies evaluated the effectiveness of interventions by measuring participants’ perceived accuracy or reliability of a claim [29, 30, 36, 37, 43, 45, 53]. Moreover, some studies did not involve the retraction of misinformation in their design, which is characteristic of the research on inoculation [14, 36, 37, 53] and literacy training interventions [43], which primarily aim at improving peoples’ ability to recognise misinformation.

### Techniques to mitigate the belief perseverance bias

The extant literature has proposed only a few techniques for mitigating BPB. Nevertheless, also techniques initially developed to mitigate other biases or reduce the negative impact of misinformation could be adapted to mitigate BPB after the retraction of misinformation. In the following, we review the most relevant techniques and develop two new techniques to mitigate BPB.

#### Counter-explanation

Anderson [22] argued that BPB might be mitigated by emphasising the plausibility of an opposite or alternative hypothesis or claim. Therefore, he introduced the so-called *counter-explanation* debiasing technique. The counter-explanation technique, applied after the retraction of misinformation, induces subjects to write causal explanations for an alternative hypothesis. In particular, the subjects are asked to imagine evidence supporting the validity of an alternative hypothesis and try to explain why this alternative hypothesis might be true. Thus, the counter-explanation technique is a particular variant of refutations.

Anderson [22], Anderson and Sechler [23] and Lord et al. [24] howed in experiments that counter-explanation mitigates BPB. Although creating causal explanations for an alternative hypothesis requires high cognitive and time efforts from misinformation recipients, we see some potential for applying the counter-explanation technique in practice (details in Sec: *Effort and practical applicability*). We, therefore, consider this technique in our study and compare its effectiveness in mitigating BPB to newly developed debiasing techniques.

#### Inoculation

Anderson [22] proposed, besides the counter-explanation technique, also the so-called *inoculation*. Similarly to the counter-explanation, Anderson’s inoculation relies on inducing subjects to write causal explanations for an alternative hypothesis. Namely, it encourages the subjects to create plausible explanations for both (or all) possible hypotheses before reading a particular piece of information. The aim is to reduce unwarranted hypothesis perseverance by showing how easily any possible hypotheses might be explained. This should mitigate BPB when the initial information is later retracted. The inoculation technique proposed by Anderson [22] is actually a sub-variant of inoculations based on McGuire’s inoculation theory, as it does not include a warning component.

Anderson [22] showed that inoculation is effective in mitigating BPB. Nevertheless, creating plausible explanations for all possible hypotheses before reading any information (including information later confirmed as accurate) requires enormous cognitive and time efforts from potential misinformation recipients. For this reason, we see limited practical applicability of the inoculation technique and, therefore, do not examine this technique further.

#### Awareness training

Hammond, Keeney and Raiffa [54, p. 55] argued that ‘[…] the best protection against all psychological traps […] is awareness. […] even if you can’t eradicate the distortions ingrained into the way your mind works, you can build tests and disciplines into your decision-making process that can uncover errors in thinking before they become errors in judgements. And taking action to understand and avoid psychological traps can have the added benefit of increasing your confidence in the choices you make’. However, according to Moven and Gaeth [55, p. 185], being aware of a potential bias is not sufficient for mitigating the bias as ‘decision makers may not recognise their own fallibility until they are personally confronted with it’, and training explicitly designed for debiasing is necessary. Concerning the confirmation bias, Nickerson [26, p. 211] suggests that ‘[…] simply being aware of the confirmation bias […] might help one both to be a little cautious about making up one’s mind quickly on important issues and to be somewhat more open to opinions that differ from one’s own’. Anderson and Lindsay [56] recommend education and training to improve society’s general reasoning ability and therewith reduce potential biases typically arising from the use of naive theories–knowledge structures with a causal or explanatory component.

Aczel, Bago, Szollosi, Foldes and Lukacs [57] studied awareness training in an experiment with the aim to initiate the exploration of debiasing techniques applicable in a real-life setting and achieve lasting improvement in decision making. Their experiment focused on ten biases, including anchoring bias, overconfidence bias and outcome bias; however, BPB was omitted. Their awareness training consisted of a general introduction to heuristics and biases and a presentation and explanation of each bias, including a real-life example and some techniques to avoid bias. Morewedge, Yoon, Scopelliti and Symborski [58] successfully applied video and game awareness training interventions to mitigate bias blind spot, confirmation, fundamental attribution error, anchoring, representativeness, and social correction biases. Shepperd, Mair and Jørgensen [59] found that a three-hour workshop to enhance awareness of a range of cognitive biases positively affected debiasing professional software developers. Also the specific up-front warning about CIE studied by Ecker et al. [9] is actually a type of awareness training as it explains CIE and demonstrates its operation in two concrete examples. Ecker et al. [9] showed that such specific warning reduces but does not eliminate the continued influence of misinformation. To the best of our knowledge, there has been no research on applying awareness training to mitigate BPB.

The inoculations and literacy training interventions reviewed in Sec: *Techniques to reduce the negative impact of misinformation* can also be considered a kind of awareness training as they aim to create resistance to misinformation by raising awareness about common manipulation techniques used to spread misinformation and techniques to identify misinformation. Considering the promising results of the latest research on inoculating against common manipulation techniques and on creating resistance to misinformation through news literacy messages using the BPB paradigm reviewed in Sec: *Techniques to reduce the negative impact of misinformation* and of the research on mitigating various biases through bias awareness training reviewed above, we hypothesise that awareness training specially designed for BPB can mitigate BPB after the retraction of misinformation.

Inoculations and literacy training interventions are commonly applied as prebunking interventions before encountering misinformation, and bias awareness training is commonly applied in advance to prevent or at least reduce the potential extent of the bias. This paper applies BPB awareness training as a debunking intervention after encountering and retracting misinformation to mitigate BPB that has occurred after the retraction of misinformation. The findings of the study by Tay et al. [14] examining the effectiveness of an inoculation intervention before and after encountering misinformation suggest that BPB awareness training could be more effective when applied as a debunking intervention rather than a prebunking one.

There is a certain similarity between our BPB awareness training in the BPB paradigm and the post-warning in the eyewitness misinformation paradigm, as they are both applied as debunking interventions. However, our BPB awareness training differs substantially in terms of content. While post-warnings include a warning about misinformation in the post-event narrative and often also discredit the post-event-narrative source, our BPB awareness training includes a warning that the initial information was misinformation, a warning about the effect of BPB and an illustration of the bias on a particular example.

#### Refutation

Refutations effectively reduced the negative impact of misinformation in several studies using the classical BPB paradigm, although these studies addressed ‘reducing the impact of misinformation’ without explicitly referring to BPB (see Sec: *Techniques to reduce the negative impact of misinformation*). Ecker, O’Reilly et al. [47] and Vraga et al. [44] showed that short refutation tweets (up to 140 characters) are more effective in reducing the negative impact of false tweet claims (up to 140 characters) on participants’ opinions than simple retractions. Swire et al. [28] showed that medium-format (about 500 characters) refutations are more effective in reducing the negative impact of short-format (one-sentence) misinformation than simple retractions. Paynter et al. [48] showed that detailed extra-long-format refutation (about 10 pages) incorporating six specific elements (source credibility, self-affirmation, social norming, warning, graphical representations, salience) is effective in reducing the impact of autism treatment myths. It is worth noting that the intervention applied by Paynter et al. [48] is not just a simple refutation but a combination of refutation and bias awareness training. It contains a detailed description of the bias called ‘illusion of causality’, its role in assessing the effectiveness of autism treatments and ways to prevent it. To the best of our knowledge, there has been no research on the effectiveness of medium-format refutations in reducing the negative impact of long-format misinformation (such as whole news articles rather than just news titles or tweets) using the classical BPB paradigm.

This paper examines the effectiveness of a medium-format refutation (about 1,000 characters) in mitigating BPB after the retraction of long-format (about 4,500 characters) misinformation. The refutation is applied by providing some specific counter-arguments against the claim of misinformation to the misinformation recipients (more details in Sec: *Debiasing techniques*). The refutation should indirectly induce causal thinking in favour of an alternative claim by the misinformation recipients. There is an analogy to Anderson’s counter-explanation technique, which induces causal thinking directly by asking the recipients of misinformation to think of and write counter-arguments. Therefore, the particular form of refutation studied in this paper is termed *counter-speech*.

## Method

### Materials

#### Misinformation, its retraction and debriefing

We manipulated participants’ *opinions on the relationship between firefighters’ attitude to risk and successfulness in their job* (shortly *the risk-attitude & success relationship*). Numerous studies confirmed that participants’ opinions on this relationship could be manipulated, and BPB could be induced in an experimental setting [16, 17, 22, 23, 25]. The experiment was presented as a *study on analytical thinking and comprehension of scientific text* to conceal its real purpose. To add credibility, we included a critical thinking scale [60], a credulity scale [61] and several tasks allegedly examining participants’ comprehension of scientific text.

We exposed participants to misinformation suggesting a positive risk-attitude & success relationship (Appendix C in S1 Appendix), which was validated in the preparatory study (Appendix B in S1 Appendix). Misinformation was about one and a half pages long and consisted in presenting an invented summary of an alleged study and invented case studies of two firefighters allegedly participating in the study.

Retraction of misinformation was done in the spirit of the study’s alleged purpose. The participants were told that the research study’s summary and the case studies had been invented to analyse people’s comprehension of scientific text and analytical thinking, and the described research study had never taken place (see Appendix C in S1 Appendix). This retraction should not be confused with the debriefing concerning the real purpose of the experiment, which we conducted at the end of the experiment (see Appendix C in S1 Appendix).

#### Debiasing techniques

We examined and compared the effectiveness of three debiasing techniques in mitigating BPB: the counter-explanation technique proposed by Anderson [22] and the counter-speech and awareness-training techniques proposed in this paper. In addition, we considered for comparison purposes a control group where no debiasing technique was applied.

The **counter-explanation (CE)** technique, based on the debiasing technique of the same name proposed by Anderson [22], comprises 1) repeating that the research study presented to the participants was invented; 2) pointing out that the opposite hypothesis might be true; 3) asking the participants to think of and write at least three arguments supporting the opposite hypothesis (i.e. counter-arguments); 4) providing an example of such a counter-argument. The exact form of the CE treatment applied in our study is provided in Appendix E in S1 Appendix.

The **counter-speech (CS)** technique proposed in this paper belongs to refutations. It comprises 1) repeating that the research study presented to the participants was invented; 2) pointing out that the opposite hypothesis might be true; 3) noting that there are several arguments supporting the opposite hypothesis; 4) providing three arguments supporting the opposite hypothesis (counter-arguments); 5) asking the participants to spend time considering the provided arguments and think of other possible arguments. The exact form of the CS treatment applied in our study is provided in Appendix E in S1 Appendix.

The **awareness-training (AT)** technique is applied in our study as a debunking bias awareness-training intervention. It comprises 1) repeating that the research study presented to the participants was invented; 2) pointing out that the invented study should therefore not influence participants’ opinion; 3) introducing BPB as a phenomenon responsible for irrational behaviour; 4) illustrating the effect of BPB on a hypothetical real-life situation; 5) warning the participants about the traps of BPB. The exact form of the AT treatment applied in our study is provided in Appendix E in S1 Appendix.

We considered a **control group (CG)** where no debiasing technique was applied as a benchmark for measuring the effectiveness of the CE, CS and AT techniques in mitigating BPB. The CG received a filler task (21-item Proactive Decision-Making Scale [62]) after the retraction of misinformation.

We hypothesised that all three tested debiasing techniques mitigate BPB, while the CG treatment does not affect BPB.

**H**_**1**_: The counter-explanation (CE) technique mitigates BPB.
**H**_**2**_: The counter-speech (CS) technique mitigates BPB.
**H**_**3**_: The awareness-training (AT) technique mitigates BPB.
**H**_**4**_: The control group (CG) treatment does not affect BPB.

#### Experimental design

**Fig 1:**
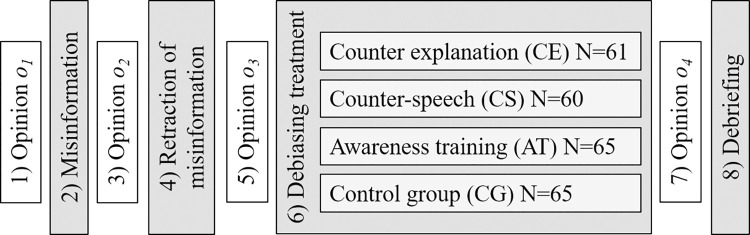
The study’s procedure with sample sizes for the debiasing treatment groups.

Our study used a *pretest–posttest between-subjects design* with three debiasing treatment groups (CE, CS and AT) and a CG. To determine changes in participants’ opinions during the experiment, identify participants with(out) BPB and compare the effectiveness of various debiasing techniques, we measured participants’ opinions four times during the experiment–at the beginning of the experiment (measurement time *t*_*1*_), after exposure to misinformation (*t*_*2*_), after exposure to misinformation retraction (*t*_*3*_) and after exposure to the debiasing treatment (*t*_*4*_) (see Fig 1). To prevent the potential practice effect and the potential effect of participants’ attempts to maintain consistency in their answers, we used different sets of measurement items at each measurement time. Furthermore, to reduce the item order effect in our study, we used *random counterbalancing*, i.e. we administered the set of measurement items to each participant in a randomly determined order. A detailed discussion of the reasons for the choice of the experimental design and the related challenges is provided in Appendix A in S1 Appendix.

#### Measures of opinion

Since this study intended to use different sets of measurement items at each measurement time, a sufficient number of such items was necessary. In the preparatory study (Appendix B in S1 Appendix), eight pairs of oppositely worded Likert items and four phi-coefficient measures (Appendix D in S1 Appendix) were validated to measure participants’ opinions on the risk-attitude & success relationship.

Likert items compared risk-taking and risk-avoiding firefighters and were assessed on a 7-point scale (1: completely disagree; 2: mostly disagree; 3: slightly disagree; 4: neither agree nor disagree; 5: slightly agree; 6: mostly agree; 7: completely agree) by selecting one answer from a dropdown list. Each phi-coefficient measure consisted of two questions related to firefighters’ attitude to risk and successfulness in their job with open text boxes where numbers between 0 and 100 had to be entered. Based on the provided numbers, the phi-coefficient was then computed (see Appendix B in S1 Appendix).

We used one phi-coefficient measure and four Likert items to measure participants’ opinions at each measurement time in the experiment. To reduce the impact of the *acquiescence bias—*the tendency to agree with statements regardless of their content [63]*—*we used the same number of positively and negatively formulated Likert items (i.e. two positively and two negatively formulated items) at each measurement time. Furthermore, we applied random counterbalancing to reduce the item order effect. The phi-coefficient measure and the Likert items were selected randomly without replacement from the set of four phi-coefficient measures and the set of eight positively and eight negatively formulated Likert items, respectively, for each participant at each measurement time. Moreover, we randomised the order of the phi-coefficient measure and Likert items and the order of the questions within the phi-coefficient measure for each participant at each measurement time.

A composite score defined on the interval scale [1,7] (1: absolutely negative risk-attitude & success relationship; 4: no risk-attitude & success relationship; 7: absolutely positive risk-attitude & success relationship) was computed at each measurement time as an average of the phi-coefficient measure (first transformed from the interval [–1,1] to the interval [1,7] by a linear transformation) and the average value of the four Likert items (the negatively formulated Likert items were first recoded). Based on the properties of the composite score, we established the threshold value for opinion change as Δ = 0.2. This means that there is a change in opinions when the difference between the composite scores at two measurement times is at least 0.2. When the difference is less than 0.2, there is no change in opinions.

#### Measuring the effectiveness of the debiasing techniques

To determine the extent to which debiasing techniques revert opinions back to the baseline and therewith mitigate BPB, opinions after exposure to debiasing techniques should be compared to initial opinions before the exposure to misinformation [64] rather than to opinions after exposure to misinformation and/or its retraction. Several studies on reducing the negative impact of misinformation have already used this way of measuring the effectiveness of interventions [8, 9, 16, 21, 65–67]. We use this approach in our experiment to assess the effectiveness of debiasing techniques in mitigating BPB after the retraction of misinformation. Specifically, this means we compare the opinions after exposure to the debiasing treatment at the measurement time *t*_*4*_ to the initial opinions at the measurement time *t*_*1*_.

### Procedure

The experimental procedure and the sample sizes for the debiasing TGs are illustrated in Fig 1. The experiment consisted of eight steps:

1. Measurement of initial opinion *o*_*1*_ (at the measurement time *t*_*1*_): Each participant completed one phi-coefficient measure and evaluated two positively and two negatively formulated Likert items, selected randomly without replacement from the set of available measures and administered in random order.
2. Misinformation: Participants were exposed to misinformation suggesting a positive risk-attitude & success relationship.
3. Measurement of opinion *o*_*2*_ after exposure to misinformation (at the measurement time *t*_*2*_): Same as step 1.
4. Retraction of misinformation: This was done in the spirit of the alleged purpose of the study.
5. Measurement of opinion *o*_*3*_ after exposure to retraction (at the measurement time *t*_*3*_): Same as step 1.
6. Debiasing treatment: Each participant was randomly assigned to one of the three debiasing TGs (CE, CS or AT) or a CG
7. Measurement of opinion *o*_*4*_ after exposure to the debiasing treatment (at the measurement time *t*_*4*_): Same as step 1.
8. Debriefing: Participants were debriefed about the real purpose of the experiment.

### Participants

The study was conducted in English, and the participants needed a high intermediate level of English to ensure high data quality. Most participants (327) were recruited by Qualtrics^©^ in the UK. Additionally, we conducted the experiment with 39 first-year business students from an English study programme at the Management Center Innsbruck. All participants gave written informed consent prior to their participation by viewing a screen with informed consent information and clicking on the “agree” button. The data were collected anonymously, and the participants were allowed to quit the experiment at any time. The study received written approval from the Ethics Committee of the Management Center Innsbruck.

An apriori power analysis for one-way ANOVA with four groups (one-way ANOVA is used to examine the effect of debiasing treatments on participants’ BPB) using Gpower showed that a total of 276 participants with BPB was required to detect a medium effect size (partial *η*^2^ = 0.06) with 1−*β* = 0.95 and *α* = 0.05. Since BPB was observed by only some participants, an accordingly higher total sample was needed. We, therefore, checked the collected data regularly (namely the number of participants with BPB and the effect size for one-way ANOVA) and stopped the collection after reaching the total sample of 366 participants, of which 251 participants showed BPB (see Sec: *Belief perseverance bias sample*).

The total sample (N = 366) consisted of 196 females and 170 males. Overall, 118 participants were aged between 18 and 23, 129 were between 24 and 29, and 119 were between 30 and 35. Furthermore, 193 participants attained a university education, 172 attained a high school education, and one did not finish high school. Moreover, 223 participants were employed, 48 were unemployed, and 95 were students. The median time spent on the study was 23.3 minutes (interquartile range IQR = 11.4).

### Belief perseverance bias sample

For analysing the effectiveness of the debiasing techniques, we only considered participants suffering from BPB. To identify and eliminate participants without BPB from the sample, we operationalised BPB as follows. First, when the participant’s opinion moves from the initial opinion at *t*_*1*_ in the direction suggested by the (mis)information at *t*_*2*_ (i.e. *o*_*2*_ ≥ *o*_*1*_ + Δ, where Δ is a given threshold value for opinion change), we say that the misinformation has biased the participant and they have a *biased opinion*. When a participant with a biased opinion perseveres in their biased opinion even after the retraction of misinformation at *t*_*3*_ (i.e. when *o*_*3*_ ≥ *o*_*1*_ + Δ), we say that the participant suffers from BPB. Based on the properties of the composite score, we established the threshold value expertly as Δ = 0.2 (see Sec: *Measures of opinion*). To summarise, participants with BPB in our study are such with *o*_*2*_ ≥ *o*_*1*_ + 0.2 and *o*_*3*_ ≥ *o*_*1*_ + 0.2.

In the total sample of 366 participants, 312 participants (85%) showed biased opinions after exposure to misinformation (i.e. *o*_*2*_ ≥ *o*_*1*_ + 0.2), while only 28 participants (8%) showed no opinion change and 26 participants (7%) changed their opinions in the opposite direction. Out of the 311 participants with biased opinions after exposure to misinformation, 251 (68.5% of the total sample) suffered from BPB after the retraction of misinformation (*o*_*3*_ ≥ *o*_*1*_ + 0.2).

The sample of N = 251 participants with BPB consisted of 138 females and 113 males. Furthermore, 85 participants were aged between 18 and 23, 83 were between 24 and 29, and 83 were between 30 and 35. Furthermore, 120 participants attained a university education, 130 attained a high school education, and one did not finish high school. Moreover, 148 participants were employed, 37 were unemployed, and 66 were students.

## Results

For analysing changes in opinions, the extent of BPB and the effectiveness of the debiasing techniques, we used a series of within-subject t-tests and one-way ANOVA with planned contrasts. Statistical analyses were performed using the Real Statistics Resource Pack for Excel.

### Biasing

**Fig 2:**
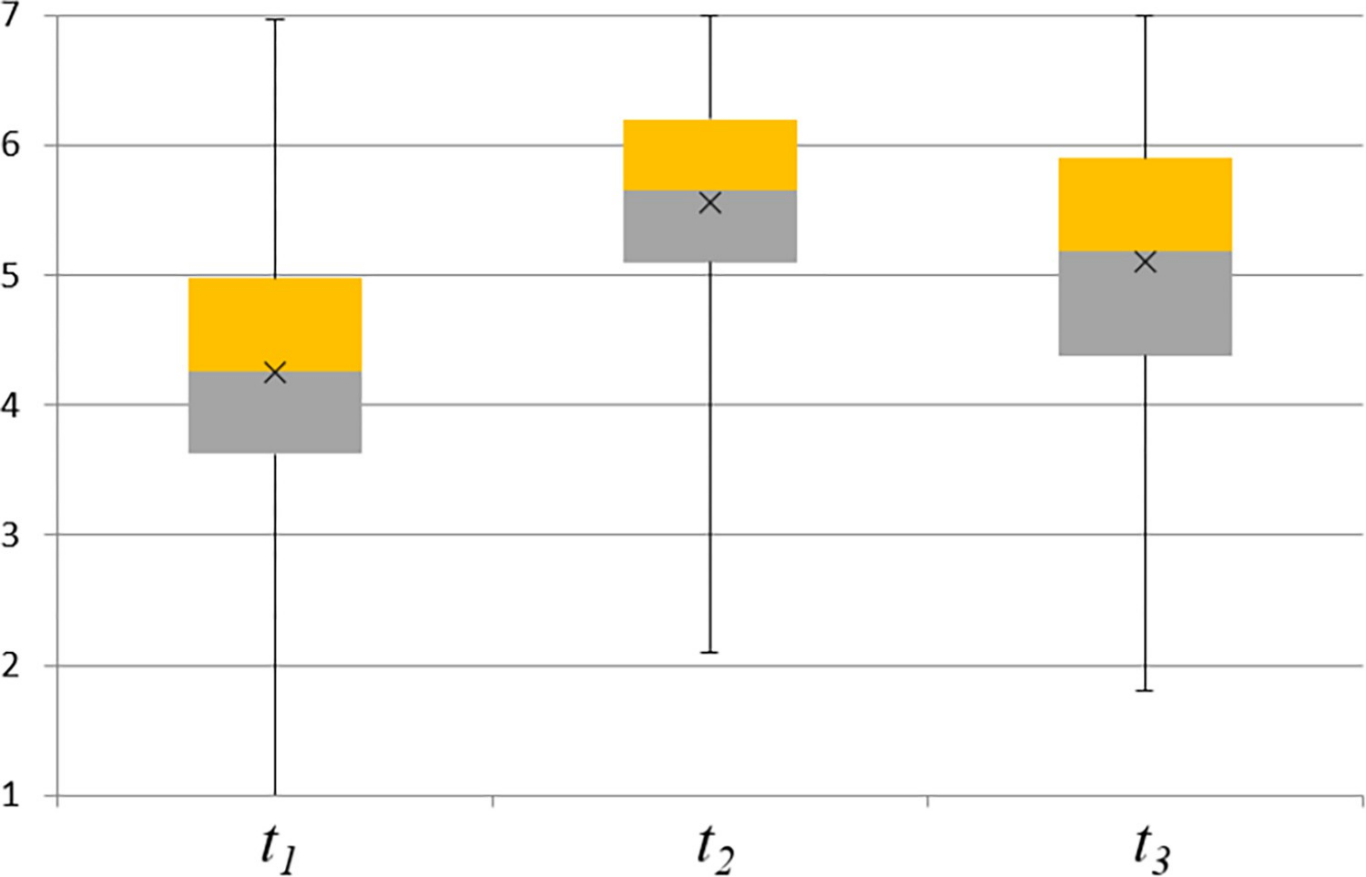
Boxplots of participants’ opinions (N = 366) at the measurement times *t*_*1*_, *t*_*2*_ and *t*_*3*_. The top of the upper whisker and the bottom of the lower whisker represent the maximum and minimum values, the top and bottom of the box represent the 75^th^ and 25^th^ percentiles, the line through the box represents the median, and the x marker represents the mean of the sample.

The participants’ mean initial opinion at *t*_*1*_ was that risk-taking firefighters are slightly more successful in their jobs than risk-avoiding firefighters (composite score on the interval scale [1,7]: M_1_ = 4.26, SD = 1.04). The participants changed their opinions in the positive direction after exposure to misinformation at *t*_*2*_ (M_2_ = 5.55, SD = 0.90), *t*_*1*,*2*_(365) = −22.99, *p* = 3E-73, Cohen’s effect size *d* = 1.20. This means that misinformation biased the participants’ opinions in the expected direction. Their opinions then moved back in the direction of their initial opinions after the retraction of misinformation at *t*_*3*_ (M_3_ = 5.10, SD = 1.08), *t*_2,3_ (365) = 8.79, *p* = 2.9E-17, *d* = 0.46. Nonetheless, their opinions at *t*_*3*_ were still significantly different from their initial opinions at *t*_*1*_, *t*_*1*,*3*_(365) = −15.26, *p* = 2.7E-41, *d* = 0.80. This result demonstrated the presence of BPB in the experimental sample. The boxplots of participants’ opinions at the measurement times *t*_*1*_, *t*_*2*_ and *t*_*3*_ are shown in Fig 2.

### Effectiveness of the debiasing techniques

**Fig 3:**
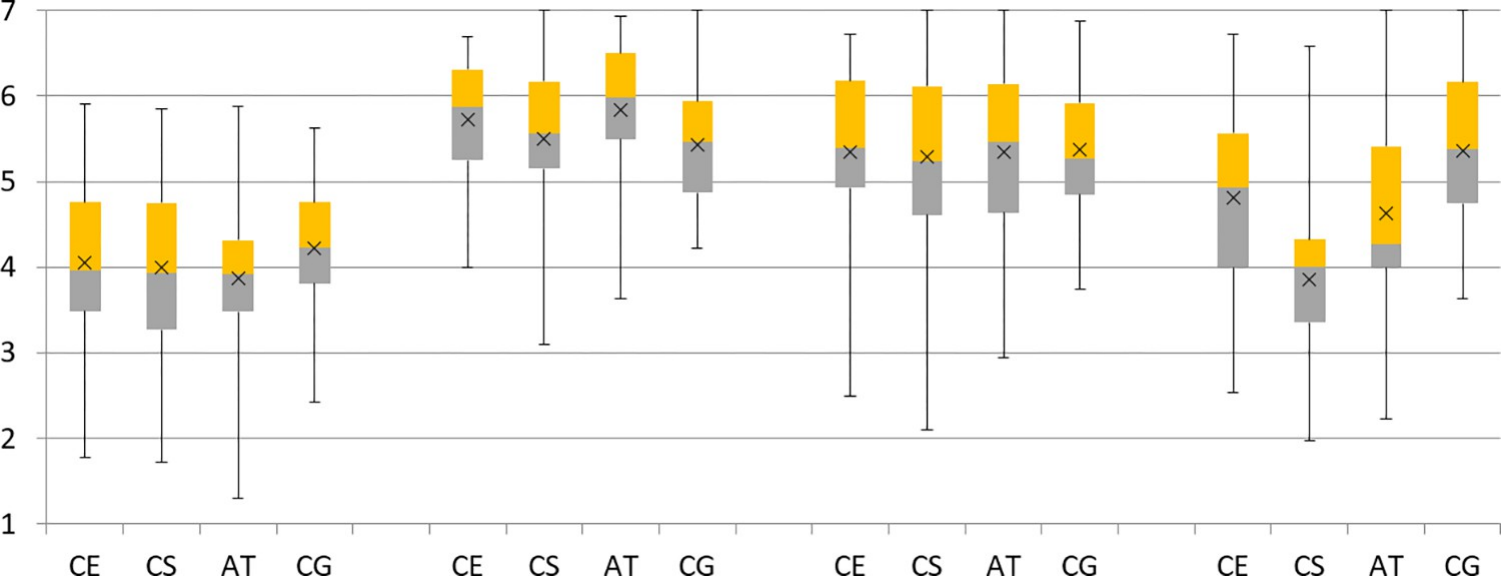
Boxplots of participants’ opinions (N = 251) for the debiasing treatment groups at each measurement time. The top of the upper whisker and the bottom of the lower whisker represent the maximum and minimum values, the top and bottom of the box represent the 75^th^ and 25^th^ percentiles, the line through the box represents the median, and the x marker represents the mean of the sample.

For assessing the effectiveness of the counter-explanation (CE), counter-speech (CS) and awareness-training (AT) techniques in mitigating BPB, we used the difference between the initial opinions at the measurement time *t*_*1*_ and the opinions after exposure to a debiasing treatment at *t*_*4*_, i.e. *o*_*1*_ *–o*_*4*_, in the sample of N = 251 participants with BPB and compared the corresponding TGs with the control group (CG). Table 1 shows the relevant statistics for the TGs and the CG at each measurement time and the differences *o*_*1*_ *–o*_*4*_. Fig 3 shows the boxplots of participants’ opinions for the debiasing TGs at each measurement time.

**Table 1:** Opinion means and standard deviations at each measurement time and t-tests for the differences *o*_1 –_ *o*_*4*_ for the debiasing treatment groups.

Group	N	Means				Standard deviations				*o*_*1*_ *–o*_*4*_				
		M_1_	M_2_	M_3_	M_4_	SD_1_	SD_2_	SD_3_	SD_4_	M_1-4_	CI_95%_	*t*-stat	p-value	Cohens’ *d*
CE	61	4.01	5.70	5.33	4.78	0.94	0.78	1.04	1.09	−0.78	[−1.01, −0.53]	−6.50	8.7E-09	0.83
CS	60	3.99	5.60	5.40	3.87	0.99	0.79	1.01	1.15	0.12	[−0.15,0.39]	0.87	0.39	0.11
AT	65	3.97	5.83	5.32	4.73	0.99	0.79	0.93	1.13	−0.76	[−1.01, −0.52]	−6.15	2.8E-08	0.76
CG	65	4.06	5.55	5.27	5.25	0.86	0.82	0.97	1.02	−1.19	[−1.40, −0.97]	−11.10	7.4E-17	1.38

The CG serves as a benchmark for analysing the effectiveness of the debiasing techniques. There was no change in opinions between the measurement times *t*_*3*_ (M_3_ = 5.27, SD = 0.97) and *t*_*4*_ (M_4_ = 5.25, SD = 1.02) in the CG, *t*(64) = 0.41, *p* = 0.68. Thus, hypothesis H_4_ was confirmed. Furthermore, the TOST equivalence test showed that the opinions at *t*_*3*_ are equivalent to the opinions at *t*_*4*_ as CI_90%_ = [−0.07,0.12] lies well within the equivalence interval [−0.2,0.2].

One-factor ANOVA on the differences *o*1 –*o*_4_ revealed a significant effect of the debiasing treatments, *F*(3,247) = 20.08, *p* = 1.1E-11, *η*^2^ = 0.20. Planned contrasts with Bonferroni correction further showed a significant reduction of the BPB for the CE treatment (*t*(247) = 2.41, *p* = 0.017), the CS treatment (*t*(247) = 7.57, *p* = 7.5E-13) and the AT treatment (*t*(247) = 2.50, *p* = 0.013) compared to the CG. Thus, hypotheses H_1_, H_2_ and H_3_ were supported, i.e. all three debiasing techniques mitigated the BPB. The effect size was medium for the CE and AT techniques (*d* = 0.43 and *d* = 0.44, respectively) and very large for the CS technique (*d* = 1.36). Table 2 shows the planned contrasts for the debiasing TGs on the differences *o*1 –*o*_4_ with the corresponding statistics.

**Table 2:** Planned contrasts with the corresponding statistics for the debiasing treatment groups on the differences *o*_*1*_ *–o*_*4*_.

Contrasts	CE	CS	AT	CG	M	CI_95%_	*t*-stat	*p*-value	Cohen’s *d*
Contrast 1	1			–1	0.41	[0.08, 0.75]	2.41	0.017	0.43
Contrast 2		1		–1	1.31	[0.97, 1.64]	7.57	7.5E-13	1.36
Contrast 3			1	–1	0.42	[0.09, 0.76]	2.50	0.013	0.44

Significance level with Bonferroni correction: α = 0.017.

Within-subject t-tests showed a significant difference between participants’ opinions at *t*_*1*_ and *t*_*4*_ for the CE and AT treatments (see Table 1). Thus the AT and CE treatments mitigated but did not eliminate BPB. Contrarily, there was no significant difference between participants’ opinions at *t*_*1*_ and *t*_*4*_ for the CS treatment (see Table 1). The equivalence of participants’ opinions at *t*_*1*_ and *t*_*4*_ for the CS treatment was not confirmed by the TOST equivalence test, as CI_90%_ = [–0.11, 0.35] did not lie within the equivalence interval [–0.2, 0.2]. Nevertheless, the CI_90%_ contained 0, which suggests that the CS technique could eliminate BPB. Moreover, the positive mean difference of participants’ opinions at *t*_*1*_ and *t*_*4*_ suggests that the CS technique could even work too strongly and push participants’ opinions in the opposite direction, although the effect size is very low.

We further compared the CE and AT techniques in terms of effectiveness. Although the TOST equivalence test did not confirm the equivalence of the CE and AT techniques in terms of their effectiveness as CI_90%_ = [–0.28, 0.29] did not lie within the equivalence interval [–0.2, 0.2], the CI_90%_ containing 0 suggested no difference in the effectiveness between the techniques.

## Discussion

This paper contributed to the research on mitigating the belief perseverance bias (BPB) after the retraction of misinformation by developing new debiasing techniques with higher practical applicability than the existing debiasing techniques. Based on the literature review, we developed two debiasing techniques and conducted a study to compare their effectiveness in mitigating BPB with an existing debiasing technique. This section discusses the results, reviews the limitations and suggests directions for future research.

### Discussion of results

We developed counter-speech (CS) and awareness-training (AT) techniques and compared them in an experiment with the counter-explanation (CE) technique proposed by Anderson [22] in terms of effectiveness in mitigating BPB. In the following, we discuss the effectiveness of the debiasing techniques and the effort related to applying the techniques in practice and derive conclusions on the practical applicability of the techniques.

#### Effectiveness

A prerequisite for examining the effectiveness of techniques to mitigate BPB after the retraction of misinformation in our study was observing BPB by the participants after retraction of misinformation. The study fulfilled this prerequisite. Namely, 85% of the participants in our study became biased by the misinformation, and 68.5% showed BPB after the retraction of misinformation. These high numbers demonstrate how easily individuals’ opinions can be influenced by misinformation and emphasise the importance of techniques for mitigating BPB.

All three tested debiasing techniques mitigated BPB after the retraction of misinformation (hypotheses H_1_, H_2_ and H_3_ were supported). The CS technique was the most effective among the three techniques with a very large effect size. The CE and AT techniques, with medium effect sizes, were close to being equivalent in terms of their effectiveness.

The CE and CS techniques were supposed to induce causal thinking by the subjects (directly by CE and indirectly by CS) in support of an alternative claim. Causal thinking has been argued as an essential determinant of BPB, and the availability of causal arguments for an alternative claim has been shown to negatively correlate with BPB [25]. The availability of causal arguments for an alternative claim might explain why the CS technique is more effective in mitigating BPB than the CE technique. Perhaps not all subjects in the CE condition were willing to spend the necessary cognitive effort to create arguments against the original claim. Additionally, despite spending reasonable cognitive effort, they may have found it difficult to create causal arguments (note that we did not measure cognitive effort and the availability of causal arguments in our study). Contrarily, providing counter-arguments to the subjects in the CS condition might have made causal arguments for an alternative claim more available, making the alternative claim more feasible in the CS condition than in the CE condition.

#### Effort and practical applicability

The **CE technique** proposed by Anderson [22] consists of asking the misinformation recipients to think of and write counter-arguments. Thus, the CE technique requires active participation from the recipients of misinformation (e.g. news consumers encountering misinformation in various media or decision makers encountering misinformation related to a specific decision problem supported by decision analysis tools), which is associated with high cognitive and time efforts compared to the CS and AT techniques. Furthermore, providers of debiasing (e.g. fact-checkers verifying claims and news in information and social media or decision analysts providing support to decision makers in specific decision problems) have to formulate the text of the CE treatment for every piece of misinformation.

Because of this, we see only limited practical applicability of the CE technique in situations where recipients frequently encounter misinformation that is not related to a specific decision problem, such as news consumers encountering misinformation in various media. In media, news consumers are likely to frequently encounter misinformation on various topics that are not necessarily of high importance to them and they may, therefore, not be willing to undergo the high cognitive and time effort related to the CE technique. Contrarily, we see some potential for the CE technique to be applied in situations where recipients encounter misinformation related to a specific decision problem of high importance to them. In particular, we can assume that decision makers encountering misinformation related to an important decision problem are willing to accept the additional effort related to the CE technique to ensure high-quality outcomes of their decisions.

In the **CS technique**, the recipients of misinformation were asked to read a text with counter-arguments. This technique requires only passive participation of the misinformation recipients and is, therefore, associated with low cognitive and time efforts compared to the CE technique. On the other hand, the CS technique requires higher effort from the providers of debiasing than the CE technique, as they must formulate the counter-arguments. The counter-arguments must be designed for every piece of misinformation or at least for every topic subject to misinformation. For example, one standardised CS text containing arguments for COVID-19 vaccinations might be used to counter misinformation containing arguments against vaccination. Despite the higher effort from the providers of debiasing, we see a high potential for practical applicability of CS to misinformation in various contexts, such as frequent misinformation spread in media or misinformation related to a specific decision problem in which a decision analyst is involved. Indeed, fact-checkers willingly spend substantial effort identifying misinformation in media, and decision analysts put effort into modelling decision problems and eliciting judgemental inputs from decision makers. Therefore, we can assume that both fact-checkers and analysts are willing to exert additional effort to apply the CS technique and enhance the effectiveness of fact-checking and the quality of the decision analysis outcomes.

Our study’s CS treatment included arguments for an alternative (or opposite) hypothesis. CS can be applied in this form only when an alternative hypothesis or explanation exists. However, there are many situations where an alternative hypothesis is unknown, even when it is clear that the initial information was incorrect [34]. In such cases, arguments for why the retracted hypothesis is not true could be provided instead.

In the **AT technique,** the recipients of misinformation were asked to read a short text explaining the BPB effect. Like the CS technique, the AT technique requires only passive participation of the recipients of misinformation and is, therefore, associated with low cognitive and time efforts compared to the CE technique. Additionally, since the general text of the AT technique does not need adaption for a particular piece of misinformation, the effort of the providers of debiasing related to the application of the AT technique is negligible. We, therefore, see a high potential of the AT technique for practical applicability.

Our study applied the AT technique as a debunking intervention after the retraction of misinformation. Since the AT text does not require adaption for a particular piece of misinformation, it is also universally applicable as a prebunking intervention before encountering misinformation, mitigating or preventing the potential BPB after the retraction of misinformation. This feature further enhances the practical applicability of the AT technique. For example, in information and social media, AT could be automatically attached to all news and claims concerning specific topics identified as susceptible to misinformation. AT on BPB could also be applied as a part of an initiative to raise awareness and improve societal resilience to misinformation.

### Limitations and future research

This study used misinformation concerning a topic used in the pioneering experiments on BPB. This topic has a particular feature, as it is supposed to be of low relevance to the experimental participants. Therefore, we can assume that most participants are not involved with this topic and have no pre-formed opinions. We assume that the participants have not been actively thinking about the topic before the experiment and that they ‘form’ their first opinions regarding the topic only at the beginning of the experiment as they are asked for their opinions. This feature might have made biasing participants’ opinions and inducing BPB in an experimental setting easier than other topics. Additionally, care should be taken when generalising conclusions about the effectiveness of the debiasing techniques in mitigating BPB after the retraction of misinformation; the effectiveness of these techniques likely depends on the topic. This study considered a topic of low relevance to the participants. Therefore, we have no information on the effectiveness of the debiasing techniques with topics of high relevance. Moreover, BPB caused by misinformation concerning topics of low relevance is likely to have only limited negative implications for individuals, organisations and society. Contrarily, BPB caused by misinformation concerning topics of high relevance (such as health, money, safety, politics or specific important decision problems modelled using decision analysis) can have profound implications. Therefore, further research on BPB should focus on misinformation concerning topics of high relevance to individuals.

The retraction of misinformation and the debiasing were done in our study shortly after reading misinformation, as it is common in studies on reducing the negative impact of misinformation and mitigating the BPB. However, in practice, the time between reading a piece of information and finding out that it was misinformation is usually longer. Therefore, future research should examine how extending the time between reading misinformation, its retraction and the application of a debiasing technique impacts the strength of BPB and the effectiveness of the debiasing.

Future research should also examine adapting the debiasing techniques to specific target groups of the recipients of misinformation (such as people with low/high education, people consuming news on social media, or decision makers dealing with specific decision problems supported by decision analysis tools). We assume that appropriately adapting the debiasing techniques, particularly the CS and AT, to the specific target group (e.g. illustrating BPB on a simple example from everyday life for people with a low level of education and on a sophisticated example for people with a high level of education) may help to increase their effectiveness.

The debiasing source is another factor that could influence the effectiveness of the debiasing techniques, particularly concerning misinformation spread in media, and should, therefore, also be examined in future research. Research has shown that retractions from highly-credible sources are more effective than those from low-credible sources [68]. Therefore, we expect that CS, which belongs to refutations, can be more effective when it comes from high- rather than low-credibility sources.

Another interesting question for future research, particularly concerning BPB with topics of high relevance, is whether and to what extent people’s existing views influence the effectiveness of the debiasing techniques.

This study applied the AT technique as a debunking intervention after the retraction of misinformation in our study. As discussed in Sec: *Effort and practical applicability*, AT is also applicable as a prebunking intervention before encountering misinformation. Since this study did not examine the effectiveness of such prebunking intervention in mitigating BPB, future research should examine whether AT on BPB, applied as a prebunking intervention, could increase the effectiveness of the retraction of misinformation in mitigating BPB or even prevent bias. There is already some evidence that this might work. Specifically, Ecker et al. [9] showed that awareness training on the CIE applied up-front mitigates the CIE of misinformation after retraction, and also the up-front awareness trainings on other biases reviewed in Sec: *Techniques to mitigate the belief perseverance bias* showed to be effective in mitigating (although not fully eliminating) the biases in experimental settings. On the other hand, some studies suggested that prebunking interventions may lead to less accurate identification of and decreased belief in accurate information [15, 69]. Future research should, therefore, examine the potential side effects of the prebunking AT on beliefs in accurate information.

We examined the effectiveness of single debiasing techniques in mitigating BPB after the retraction of misinformation. However, we did not determine whether combining various techniques could increase their effectiveness in mitigating BPB. Our follow-up paper, which is currently under preparation, examines, among others, the effectiveness of combined AT and CS techniques.

## Conclusions

The belief perseverance bias (BPB) makes individuals persevere in their beliefs and opinions even after the misinformation on which they were based has been retracted. In this way, misinformation can negatively impact individuals’ beliefs, opinions and consequently decisions. Despite the high relevance of BPB, only a few debiasing techniques with low practical applicability were introduced.

This paper has contributed to the research on reducing the negative impact of misinformation on individuals’ opinions by developing techniques to mitigate BPB after the retraction of misinformation. In developing the debiasing techniques, we focused on practical applicability, drawing from the existing literature on reducing the negative impact of misinformation and mitigating other biases. As a result, we proposed two new techniques for mitigating BPB—counter-speech (CS) and awareness training (AT). We compared the debiasing techniques with the existing counter-explanation (CE) technique in terms of effectiveness in an experiment using the classical BPB paradigm. Furthermore, we discussed the effort of the providers and recipients of debiasing related to the use of the debiasing techniques and their applicability in practice.

All three debiasing techniques—CE, CS and AT—mitigated BPB after the retraction of misinformation in our study. The CS technique was more effective in mitigating BPB than the AT and CE techniques, which were close to being equivalent in terms of their effectiveness. The CS and AT techniques are associated with less cognitive and time effort of the recipients of debiasing than the CE technique. On the other hand, the AT and CE techniques require less effort from the providers of debiasing than the CS technique. Therefore, we recommend that the providers of debiasing use the CS technique as the first choice to achieve the best debiasing effect. Contrarily, the AT technique should be used when there is a need to keep the effort of the recipients and providers of debiasing low. Another advantage of the AT technique besides the low effort required from the providers and recipients of debiasing is that it is universally applicable as a prebunking intervention before encountering misinformation. In this form, AT could be used to raise awareness of BPB and improve societal resilience to misinformation.

This study has some limitations. The retraction of misinformation and the debiasing were done shortly after reading misinformation, as is common in studies on reducing the negative impact of misinformation and mitigating BPB. Therefore, it is unclear whether or to what extent the effectiveness of the debiasing techniques would change in practice, where the time between reading a piece of information and discovering that it was misinformation is typically longer. Moreover, the effectiveness of the debiasing techniques was studied on a topic of low relevance to individuals. Thus, care should be taken when generalising the conclusions about their effectiveness to other topics, particularly topics of high relevance to individuals.

The paper provides several directions for future research. First, the impact of the time between encountering misinformation, its retraction and debiasing on the effectiveness of the debiasing techniques should be studied. Second, the effectiveness of the debiasing techniques should be examined on topics of high relevance to individuals. Third, the focus should be on enhancing the applicability of the debiasing techniques in practice, particularly with misinformation spread to a broad audience through information and social media. Fourth, research should focus on enhancing the effectiveness of debiasing, e.g. by combining various debiasing techniques. Fifth, the effectiveness of the AT technique applied as a prebunking intervention should be examined and compared to the debunking AT intervention. Finally, it should be examined whether and to what extent the effectiveness of the debiasing techniques can be influenced by the source of debiasing, peoples’ existing views and opinions and adapting the debiasing techniques to specific target groups of the recipients of misinformation.

## Supporting information

S1 Appendix Supplemental material. (DOCX)
