# Supplementary material for: Effective mitigation of the belief perseverance bias after the retraction of misinformation: Awareness training and counter-speech
Source: PLoS One. 2023 Mar 8;18(3):e0282202. doi: 10.1371/journal.pone.0282202 (PMC9994702; doi:10.1371/journal.pone.0282202)
Supplement: S1 Appendix — (DOCX) [file pone.0282202.s001.docx]

# Appendix

**Effective mitigation of the belief perseverance bias after the retraction of misinformation:**

**Awareness training and counter-speech**

Jana Siebert^1^, Johannes Ulrich Siebert^2^*

^1^ Department of Applied Economics, Faculty of Arts, Palacky University Olomouc, Olomouc, Czech Republic

^2^ Department of Business and Management, Management Center Innsbruck, Innsbruck, Austria

* Corresponding author, [johannes.siebert@mci.edu](mailto:johannes.siebert@mci.edu)

[**Appendix A: Experimental design**](#_Appendix_A:_Experimental)

[**Appendix B: Preparatory study**](#_Appendix_B_-)

[**Appendix C: Misinformation, retraction of misinformation and debriefing**](#_Appendix_C–_Biasing)

[**Appendix D: Measures of opinions**](#_Appendix_D_-)

[**Appendix E: Debiasing techniques**](#_Appendix_E_-)

# Appendix A: Experimental design

Experiments on mitigating the belief perseverance bias (BPB) usually consist of three main steps: 1) manipulation of participants’ opinions on a specific topic, 2) retraction of misinformation and 3) application of a debiasing technique to mitigate BPB. The pioneering experiments on inducing and mitigating BPB by Anderson and colleagues used manipulation of participants’ opinions on the relationship between firefighters’ attitude to risk and successfulness in their job. Indeed, numerous studies confirmed that participants’ opinions on this relationship could be manipulated and BPB induced in an experimental setting (Anderson, 1982, 1983; Anderson, Lepper, & Ross, 1980; Anderson, New, & Speer, 1985; Anderson & Sechler, 1986). We, therefore, adopted this topic for our study.

In studies on BPB, the posttest*–*only control group design has usually been used (Anderson et al., 1980; Anderson, 1982, 1983). However, this design does not allow for determining whether there is a difference between the experimental and control groups before the study, and, more importantly, it does not allow for identifying participants who show BPB after the retraction of misinformation. To overcome these drawbacks, we use the pretest-posttest control group design in our study. In order to be able to determine changes in participants’ opinions during the experiment and thus identify participants with(out) BPB and compare the effectiveness of various debiasing techniques, we measure participants’ opinions four times during the experiment.

The same measurement items are commonly used for pretest and posttest in pretest-posttest designs (with or without a control group). For example, Maegherman, Ask, Horselenberg, and van Koppen (2021) used the same set of items three times within a study on BPB, and Roozenbeek and van der Linden (2019) used the same sets of items for the pretest and posttest in their study to test the effectiveness of the inoculation game Bad News in increasing resistance to online misinformation. However, repeatedly using the same set of measurement items within an experiment may negatively impact the results. For example, presenting the same set of items twice in experiments in which participants’ performance is tested (such as the ability to spot fake news in the experiment by Roozenbeek and van der Linden (2019)) may cause a practice effect (participants improve in the posttest after having ‘practiced’ on the same items in the pretest). Contrarily, presenting the same set of items twice in experiments in which participants’ opinion or belief is measured (such as in experiments on BPB) may lead to no significant results as the participants are likely to try to maintain consistency (at least to some degree) in their answers. Indeed, Maegherman et al. (2021) failed to observe BPB in their experiment, which might be because they used the same sets of items three times during the experiment. To overcome these problems, we use different sets of measurement items at each measurement time in our experiment.

Nevertheless, using different sets of items for the pretest and posttest is related to another problem – the *item order effect*. This means that the order of the sets of items might influence the results of an experiment. Roozenbeek, Maertens, McClanahan, and van der Linden (2021) examined the item order effect in the experiment on the effectiveness of the Bad News game conducted by Roozenbeek and van der Linden (2019). They found a significant effect for one order and no effect for the other order of two sets of items. To reduce the item order effect in our study, we use random counterbalancing - administering the set of measurement items to each participant in a randomly determined order.

The repeated measurement of participants’ opinions in our study requires a relatively large number of measurement items suitable for measuring opinions on the topic. These items must be first developed and validated. Further, since we intend to use a new treatment to manipulate participants’ opinions in our study, the suitability of such treatment for biasing participants’ opinions should be tested first. Therefore, we conducted a preparatory study (Appendix B), in which we developed and validated two biasing treatments and numerous items for measuring participants’ opinions on the topic. Afterwards, we used one validated biasing treatment and a set of validated measurement items in the main study to examine the effectiveness of two new debiasing techniques in mitigating BPB and compare them to an existing debiasing technique.

**References**

Anderson, C. A. (1982). Inoculation and counterexplanation: Debiasing techniques in the perseverance of social theories. *Social Cognition*, *1*(2), 126–139.

Anderson, C. A. (1983). Abstract and concrete data in the perseverance of social theories: When weak data lead to unshakeable beliefs. *Journal of Experimental Social Psychology*, *19*(2), 93–108.

Anderson, C. A., Lepper, M. R., & Ross, L. (1980). Perseverance of social theories: the role of explanation in the persistence of discredited information. *Journal of Personality and Social Psychology*, *39*(6), 1037–1049.

Anderson, C. A., New, B. L., & Speer, J. R. (1985). Argument Availability as a Mediator of Social Theory Perseverance. *Social Cognition*, *3*(3), 235–249.

Anderson, C. A., & Sechler, E. S. (1986). Effects of explanation and counterexplanation on the development and use of social theories. *Journal of Personality and Social Psychology*, *50*(1), 24–34.

Maegherman, E., Ask, K., Horselenberg, R., & van Koppen, P. J. (2021). Law and order effects: On cognitive dissonance and belief perseverance. *Psychiatry, Psychology and Law*, 1–20.

Roozenbeek, J., Maertens, R., McClanahan, W., & van der Linden, S. (2021). Disentangling Item and Testing Effects in Inoculation Research on Online Misinformation: Solomon Revisited. *Educational and Psychological Measurement*, *81*(2), 340–362.

Roozenbeek, J., & van der Linden, S. (2019). Fake news game confers psychological resistance against online misinformation. *Palgrave Communications*, *5*(1), 1–10.

# Appendix B: Preparatory study

The preparatory study aims 1) to develop a biasing treatment and confirm its suitability for biasing participants’ opinions on the relationship between firefighters’ attitude to risk and successfulness in their job (shorty a *risk-attitude & success relationship*) in an experimental setting and 2) to develop and validate measures of participants’ opinions on the risk-attitude & success relationship.

## Method

### Participants

The participants were recruited by Qualtrics^©^. The study was conducted in English, and the participants needed a high intermediate level of English to ensure high data quality. All participants gave written informed consent prior to their participation by viewing a screen with informed consent information and clicking on the “agree” button. The data were collected anonymously, and the participants were allowed to quit the experiment at any time. The study received written approval from the Ethics Committee of the Management Center Innsbruck.

An apriori power analysis for paired t-tests (paired t-tests are used to study the effect of two biasing treatments on participants’ opinions, see *Validation of the biasing treatments* in this appendix) using Gpower showed that a total of 90 participants was required to detect a medium effect size (d=0.5) with 1−*β* = 0.95 statistical power and *α* = 0.05 probability of type I error. We collected data from 92 participants.

The total sample (N = 92) consisted of 41 females and 51 males, 51 residing in the UK, 27 in the Netherlands, and 14 in Germany. Overall, 32 participants were aged between 18 and 23, 30 participants were between 24 and 29 and 30 participants were between 30 and 35. Furthermore, 43 participants attained a university education, 48 attained a high school education, and one did not finish high school. Moreover, 48 participants were employed, 11 were unemployed, 29 were students and 4 had another type of occupation. The median time spent on the study was 24.4 minutes (IQR = 11.0).

### Materials

#### Biasing treatment

One purpose of the preparatory study was to develop a biasing treatment and confirm its suitability for biasing participants’ opinions and inducing BPB in an experimental setting. We designed two biasing treatments: one treatment suggesting a positive risk-attitude & success relationship (i.e. suggesting that risk-taking firefighters are more successful in their job than risk-avoiding firefighters), the other treatment suggesting a negative risk-attitude & success relationship (i.e. suggesting that risk-avoiding firefighters are more successful in their job than risk-taking firefighters). Each treatment consisted in presenting 1) an invented summary of an alleged research study suggesting either a positive or negative risk-attitude & success relationship and 2) invented case studies of two firefighters allegedly participating in the study (see Appendix C).

The experiment participants were randomly assigned to one of two treatment groups (shortly TG). One TG (N = 48) received the biasing treatment suggesting a positive risk-attitude & success relationship (shortly a *positive treatment* and a *positive TG*). The other TG (N = 44) received the biasing treatment suggesting a negative risk-attitude & success relationship (shortly a *negative treatment* and a *negative TG*).

#### Measures of opinion

Another purpose of the preparatory study was to develop and validate measures of participants’ opinions on the risk-attitude & success relationship. For this purpose, we adopted (with slight modifications) one measure proposed by Anderson (1982) and developed four additional types of measures based on direct comparisons, Likert items, phi coefficients and pairwise comparison matrices. Each type of measure is described in more detail below, except the measure based on pairwise comparison matrices, as this measure was later not used for the main study.

**Slider**

We used, with slight modifications, the measure originally used by Anderson (1982). In particular, we asked the participants to indicate their opinion on the risk-attitude & success relationship on a slider scale ranging from −100 to 100 (−100: absolutely negative relationship; 0: no relationship; 100: absolutely positive relationship) with the slider anchored at the mid-point 0 of the scale. Note that Anderson (1982) used a scale ranging from −50 (highly negative relationship) to 50 (highly positive relationship). From now on, we will shortly refer to this measure as the *slider*.

**Direct comparison**

The most obvious way to get participants’ opinions on the risk-attitude & success relationship is to ask them directly. Thus, we have created two oppositely worded incomplete direct-comparison statements about the successfulness of firefighters (‘In my opinion, risk-taking firefighters tend to be ___ risk-avoiding firefighters.’ and ‘In my opinion, risk-avoiding firefighters tend to be ___ risk-taking firefighters.’) that were to be completed by choosing from the list of 9 items (1: extremely less successful than; 5: as successful as, 9: extremely more successful than). We randomly assigned one of the statements to each participant.

The direct-comparison measure (either of the two formulations) is a valid measure of participants’ opinions on the risk-attitude & success relationship. If participants’ opinions were measured only once within an experiment, the direct-comparison measure would be sufficient. However, since we intend to measure participants’ opinions several times during our experiment and use different sets of measures at each measurement time (see Appendix A), we need more measures. Therefore, we use the direct-comparison measure in this study as a reference measure for validating other measures of participants’ opinions.

**Likert items**

We created a list of nine oppositely worded Likert items about firefighters (see Appendix D) to be assessed on a 7-point scale (1: completely disagree; 2: mostly disagree; 3: slightly disagree; 4: neither agree nor disagree; 5: slightly agree; 6: mostly agree; 7: completely agree). By the opposite wording of the Likert items, we mean here that one Likert item compares risk-taking firefighters with risk-avoiding firefighters (we will shortly call such Likert item *a positively formulated (Likert) item*), while the other Likert item compares risk-avoiding firefighters with risk-taking firefighters (shortly *a negatively formulated (Likert) item*). Each Likert item was presented to participants in the form of a dropdown list with seven answer options.

**Phi coefficients**

Anderson, Lepper, and Ross (1980) and Anderson (1982) used the measures ‘new items’ and ‘criterion validity’ in their experiment. The ‘new items’ measure consisted in computing the intensity of the risk-attitude & success relationship as a simple difference (X% – Y%) of participant’s estimations of percentages of successful (denoted as X %) and unsuccessful (denoted as Y %) firefighters advising the risky option in a hypothetical item of the Risky-Conservative Choice test. Similarly, also the measure ‘criterion validity’ consisted in computing the intensity of the risk-attitude & success relationship as the difference (X% – Y%) of participant’s estimations of the percentage of risky responses of successful firefighters (X%) and the percentage of risky responses of unsuccessful firefighters (Y%) in the Risky-Conservative Choice test. However, it is not clear how this simple difference should represent the intensity of the risk-attitude & success relationship.

The intensity of the risk-attitude & success relationship can be better described using the *phi coefficient* (sometimes called the mean square contingency coefficient), which is frequently used in statistics to measure the intensity of the relationship between two binary variables. The phi coefficient reaches values between −1 and 1, with 0 representing no relationship between the variables and -1 and 1 representing perfect negative and perfect positive relationship between the variables, respectively.

Using the values X and Y above, the phi coefficient for the intensity of the risk-attitude & success relationship is given as

$$\phi=\frac{X-Y}{\sqrt{\left( X+Y \right)(200-X-Y)}} .$$

Positive values of $\phi$ represent a positive risk-attitude & success relationship, while negative values of $\phi$ represent a negative risk-attitude & success relationship. The bigger the absolute value of $\phi$ is, the stronger the intensity of the relationship is.

We created a list of four phi-coefficient measures (see Appendix D). Each measure consisted of two questions related to firefighters’ attitude to risk and successfulness in their job with open text boxes where participants were required to provide numbers between 0 and 100. The phi coefficient was then computed from the provided numbers.

**Process of validation of the measures**

The process of developing and validating the measures of opinion consisted of four steps. In the first step, we proposed four types of measures for measuring participants’ opinions: direct comparisons, Likert items, phi coefficients and pairwise comparison matrices.

In the second step, we assessed the suitability of all four types of measures for measuring participants’ opinions in collaboration with 18 experts. The experts were active participants in the 2019 Workshop of the Working Group ‘Decision Theory and Practice’ of the German Society for Operations Research. We presented the research project, described the four types of measures and distributed questionnaires to the experts. The questionnaires contained a brief description of each type of measure, a particular example of the measurement item as it would appear in the experiment and two questions regarding the understandability and the validity of the given type of measure. Namely, the experts were asked to assess a) whether the (type of) measure (the task behind it to be completed by the experiment participants) is for the participants easy or difficult to understand and b) whether it measures what it is supposed to measure. Afterwards, we discussed the pros and cons of all four types of measures. Most experts agreed on the suitability of the measures based on direct comparisons, Likert items and phi coefficients. Contrarily, most experts held the opinion that the measures based on pairwise comparison matrices are too complicated for participants and not reliable. Therefore, we abandoned the measures based on pairwise comparison matrices and considered only the measures based on direct comparisons, Likert items and phi coefficients.

In the third step, we created a list of oppositely worded Likert items and a list of phi-coefficient measures and administered them to three experts for content validation. A final set of nine pairs of Likert items and four phi-coefficient measures (see Appendix D) was chosen based on their feedback.

In the fourth step, the measures were empirically validated. The set of nine pairs of Likert items and four phi-coefficient measures was administered to the participants together with one randomly chosen direction-comparison measure and the slider. The order of the measures and questions within each phi-coefficient measure was randomized for each participant to reduce the question order bias. Correlation analysis was performed to assess the concurrent validity of the Likert items and phi-coefficient measures.

### Procedure

The experimental procedure and the sample sizes for the TGs are illustrated in Figure 1. The experiment consisted of six steps:

1. Measurement of initial opinion *o_1_* (at the measurement time *t_1_*): At the beginning of the experiment, each participant completed one randomly chosen direct-comparison measure and the slider measure. The measures were administered to each participant in random order.
2. Manipulation – biasing treatment: The participants were randomly assigned to one of the two biasing TGs and received either a positive or negative treatment (i.e. a biasing treatment suggesting either a positive or negative risk-attitude & success relationship).
3. Measurement of opinion *o_2_* after biasing (at the measurement time *t_2_*): Same as step 1.
4. Validation of the measures: The participants in both TGs completed nine pairs of oppositely worded Likert items and four phi-coefficient measures (see Appendix D). The order of the measurement items and questions within each phi-coefficient measure was randomized for each participant to reduce the question order bias.
5. Debriefing: The participants were fully debriefed about the real purpose of the experiment, i.e. they were told that the research report and the case studies had been invented and that the alleged research study had never occurred.
6. Measurement of opinion *o_3_* after debriefing (at the measurement time *t_3_*): Same as step 1.

Figure 1: Preparation study’s procedure with sample sizes for the biasing treatment groups.


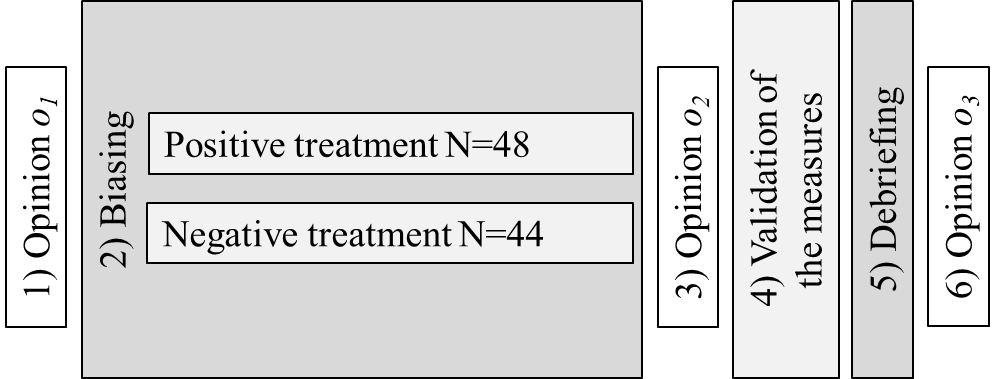


## Results and discussion

### Validation of the biasing treatments

The participants’ mean initial opinion at *t_1_* was that risk-taking firefighters are slightly more successful in their job than risk-avoiding firefighters (direct-comparison measure on the 9-point ordinal scale at *t_1_*: N = 92, M_1_= 5.83, SD = 1.53). The formulation of the direct-comparison statements had no significant effect on the answer (positive formulation (N = 50): M_1_= 5.80, SD = 1.62; negative formulation (N = 42): M_1_= 5.86, SD = 1.44), *t*(90) = 0.18, *p* = 0.86, CI_95%_= [−0.70,0.58]. The participants in the positive TG (N = 48) then changed their opinion in the positive direction at *t_2_* (M_2_= 7.15, SD = 2.14), *t_1,2_*(47) = −4.00, *p* = 1.1E-4, Cohen’s effect size *d* = 0.58. The participants in the negative TG (N = 44) changed their opinion in the negative direction at *t_2_* (M_2_= 2.34, SD = 1.52), *t_1,2_*(43) = 12.66, *p* = 2.1E-16, *d* = 1.91. Thus, we can conclude that both biasing treatments biased participants’ opinions in the desired direction.

Table 1: Sample sizes, opinion means and standard deviations for the treatment groups at the measurement times *t_1_*, *t_2_* and *t_3_*.

| Treatment group | N | Opinion means | | | Standard deviations | | |
| --- | --- | --- | --- | --- | --- | --- | --- |
|  |  | M_1_ | M_2_ | M_3_ | SD_1_ | SD_2_ | SD_3_ |
| Positive TG | 48 | 5.69 | 7.15 | 6.35 | 1.67 | 2.14 | 1.59 |
| Negative TG | 44 | 5.98 | 2.34 | 4.07 | 1.37 | 1.52 | 1.70 |
| all | 92 | 5.83 | - | - | 1.53 | - | - |

Participants’ opinions then moved back in the direction of their initial opinion after the debriefing at *t_3_* in the positive TG (M_3_= 6.35, SD = 1.59) as well as in the negative TG (M_3_= 4.07, SD = 1.70). The change in opinion was significant for both the positive TG (*t*_2,3_(47) = 2.10, *p* = 0.02, *d* = 0.30) and the negative TG (*t_2,3_*(43) = −4.95, *p* = 6E-6, *d* = 0.75). Nevertheless, the participants demonstrated BPB. Namely, their opinion at *t_3_* still significantly varied from their initial opinion at *t_1_* in the positive TG (*t_1,3_*(47) = −2.36, *p* = 0.011, *d* = 0.34) as well as in the negative TG (*t_1,3_*(43) = 6.79, *p* = 1.3E-8, *d* = 1.02). The experiment thus confirmed the suitability of both biasing treatments for biasing participants’ opinions and inducing BPB in an experimental setting. Sample sizes, opinion means and standard deviations for the treatment groups at the measurement times *t_1_*, *t_2_* and *t_3_* are shown in Table 1.

### Validation of the measures

To assess the concurrent validity of the slider, Likert items and phi-coefficient measures, correlations of the measures with the direct-comparison measure at the measurement time *t_2_* were analyzed (the negatively formulated Likert items were first recoded). Since the scales for the direct-comparison measure and the Likert items are ordinal, we applied Spearman’s coefficient *ρ*.

The correlation analysis showed strong correlations of the direct-comparison measure with the phi-coefficient measures and most Likert items. In particular, except for one pair of oppositely worded Likert items LIK_9P_ and LIK_9N_ (0.43 < *ρ* < 0.49, *p* < 2E-5) and the slider measure (*ρ* = 0.54, *p* = 3.2E-8), the correlations of all other measures with the direct-comparison measure were strong, ranging from 0.67 to 0.80 (M = 0.72, SD = 0.04, *p* < 5.5E-13). It is also worth mentioning that the correlations of the Likert items LIK_9P_ and LIK_9N_ and the slider measure with all other measures were at most moderate (0.37 < *ρ* < 0.69, *p* < 4E-4, M = 0.57, SD = 0.07) and the correlations of the slider measure with the direct-comparison measure at *t_1_* (*ρ* = 0.31, *p* = 0.003) and *t_3_* (*ρ* = 0.42, *p* = 3.2E-5) were only weak. By removing the Likert items LIK_9P_ and LIK_9N_ and the slider measure from the set of measures, the correlations among the remaining measures at *t_2_* were strong, ranging from 0.65 to 0.98 (M = 0.80, SD = 0.06).

The correlation analysis showed concurrent validity of eight pairs of Likert items and all four phi-coefficient measures. These were, therefore, adopted as valid measures of participants’ opinions on the risk-attitude & success relationship to be used in the main study.

References

Anderson, C. A. (1982). Inoculation and counterexplanation: Debiasing techniques in the perseverance of social theories. *Social Cognition*, *1*(2), 126–139.

# Appendix C: Misinformation, retraction of misinformation and debriefing

## Misinformation suggesting a positive risk-attitude & success relationship (Positive treatment)

**Title of the study: Risk-taking firefighters are more successful in their job than risk-avoiding firefighters**

**Summary:** The research study conducted at an unnamed European university was concerned with the relationship between firefighters’ attitude to risk and the successfulness of firefighters in their job. The study subjects were 126 firefighters at the end of their probationary period. The successfulness of the firefighters was evaluated by their superiors at the end of the probationary period. The firefighters completed a written risk-attitude test assessing their attitude to risk. The test contained hypothetical real-life situations in which the firefighters were asked to choose either a risk-taking or a risk-avoiding option and justify their choice. The study found a strong positive relationship between risk-taking and successfulness, i.e. the more risk-taking attitude firefighters have, the more successful they are in their job.

To give you a more precise idea about the research study, **case studies of two firefighters (one successful and one unsuccessful)** participating in the study are shown in the following. Each case study provides information on the successfulness of the respective firefighter in his job, an assessment of the firefighter’s attitude to risk based on the written risk-attitude test and one representative item from the test together with the answer given by the firefighter.

Please read both case studies carefully.

**Case study 1 – Risk-taking successful firefighter Max**

Max is considered to be a very successful firefighter. At the end of the probationary period, his performance as a firefighter was rated by his superior in the top 10% of 37 firefighters in the fire station he has been working in.

In the risk-attitude test, Max chose 18 risk-taking options and 2 risk-avoiding options. His risk attitude was thus evaluated as very risk-taking. Below is given one representative item from the risk-attitude test together with Max’s answer.

*Test item:*

Mr. R. lives in a small town in New Mexico, 150 km from the nearest hospital and doctor. His wife, 7 months pregnant, has just got severe labour contractions, and it seems that the baby is going to be born very shortly, almost two months early. Upon leaving for the hospital, Mr. R. notices that he is low on gas. He is sure that if he drives slowly, there is enough gas to get them to the hospital. However, by driving slowly, the baby is very likely to come before arriving at the hospital. On the other hand, if he drives fast, there is the danger that he runs out of gas before getting to the hospital.

Imagine that you are in Mr. R. situation. Would you take the risky decision and drive fast, hoping not to run out of gas? Or would you take the risk-avoiding decision and drive slowly, making sure that you do not run out of gas?

*Max’s answer:*

I would take the risk and drive fast to the hospital because delivering the baby in the seventh month outside the hospital is just too dangerous. So, he should not gamble with the life of their baby by driving slowly to make sure that they do not run out of gas but letting his wife deliver in the car on the way to the hospital. In case they should run out of gas before reaching the hospital, this is likely to happen not very far from the hospital anyway. And because hospitals always lie in populated areas, they can be sure to encounter other cars on the road that would immediately drive them to the hospital. So it is quite sure that they get to the hospital fast even if they should run out of gas.

**Case study 2 – Risk-avoiding unsuccessful firefighter Thomas**

Thomas is in his job as a firefighter very unsuccessful. At the end of the probationary period, his performance as a firefighter was rated by his superior in the bottom 15% of his team of 28 firefighters.

In the risk-attitude test, Thomas chose 17 risk-avoiding options and 3 risk-taking options. His risk attitude was thus evaluated as very risk-avoiding. Below is given one representative item from the risk-attitude test together with Thomas’ answer.

*Test item:*

Mr. J. is an American captured by the enemy in World War II and placed in a prisoner-of-war camp. Conditions in the camp are extremely bad, with long hours of hard physical labour and a barely sufficient diet. After spending several months in this camp, Mr. J. notes a quite promising possibility of escape by concealing himself in a supply truck that shuttles in and out of the camp. Of course, there is no guarantee that the escape would prove successful. Possible recapture by the enemy could well mean execution.

What would you advise Mr. J. to do? To take the risk and attempt to escape or to avoid the risk and stay in the camp?

*Thomas' answer:*

I would advise Mr. J. not to risk and stay in the camp. Although the possibility of escape seems to be promising, there is still a danger that he would be recaptured and executed. And since he had survived in the camp for several months already, his chances of surviving the camp until the end of the war are in my opinion not that hopeless.

## Misinformation suggesting a negative risk-attitude & success relationship (Negative treatment)

**Title of the study:** **Risk-avoiding firefighters are more successful in their job than risk-taking firefighters**

**Summary:** The study was concerned with the relationship between firefighters’ attitude to risk and their successfulness in their job. The successfulness of each firefighter in the study was evaluated based on yearly evaluation reports of their performance provided by their superiors; each firefighter was classified as ‘successful’ or ‘unsuccessful’. The firefighters completed a written risk-attitude test assessing their attitude to risk. The test contained hypothetical real-life situations in which the firefighters were asked to choose either a risk-avoiding or a risk-taking option and justify their choice. The study found a strong negative relationship between risk-taking and successfulness, i.e. that risk-avoiding firefighters are more successful in their job than risk-taking firefighters.

To give you a more precise idea about the study, case studies of two firefighters (one successful and one unsuccessful) participating in the study are shown in the following. Each case study provides information on the successfulness of the respective firefighter in his job, an assessment of the firefighter’s risk attitude based on the risk-attitude test and an example of one typical item from the test together with the answer given by the firefighter.

Please read both case studies carefully.

**Case study 1 – Risk-avoiding successful firefighter Max**

Max is considered to be a very successful firefighter. At the end of the mandatory two-year probationary period, his performance as a firefighter was rated by his superior in the top 10% of 42 firefighters in the fire station he has been working in.

In the risk-attitude test, Max chose 16 risk-avoiding options and 4 risk-taking options. His risk attitude was thus evaluated as risk-avoiding. Below is given one item from the risk-attitude test together with Thomas’s answer. Below is given one item from the risk-attitude test together with Max’s answer.

*Test item:*

Mr. R. lives in a small town in New Mexico, 150 km from the nearest hospital and doctor. His wife, 7 months pregnant, has just got severe labour contractions, and it seems that the baby is going to be born very shortly, almost two months early. Upon leaving for the hospital, Mr. R. notices that he is low on gas. He is sure that if he drives slowly, there is enough gas to get them to the hospital. However, by driving slowly, the baby is very likely to come before arriving at the hospital. On the other hand, if he drives fast, there is the danger that he runs out of gas before getting to the hospital.

Imagine that you are in Mr. R. situation. Would you take the risky decision and drive fast, hoping not to run out of gas? Or would you take the risk-avoiding decision and drive slowly, making sure that you do not run out of gas?

*Max’s answer:*

I would recommend the risk-avoiding alternative and drive slowly making sure that they do not run out of gas. He cannot know when the baby is going to be born. There are good chances that they will make it to the hospital before the baby is born. On the other hand, if the baby is born in the car before getting to the hospital, they are still going to get to the hospital to get medical care. If he drove fast and ran out of gas in the middle of nowhere, the woman would have to deliver in the car, and the baby would very likely not get the necessary medical care in time.

**Case study 2 – Risk-taking unsuccessful firefighter Thomas**

Thomas is in his job as a firefighter unsuccessful. At the end of the mandatory two-year probationary period, his performance as a firefighter was rated by his superior in the bottom 20% of his team of 30 firefighters.

In the risk-attitude test, Thomas chose 17 risk-taking options and 3 risk-avoiding options. His risk attitude was thus evaluated as risk-taking. Below is given one item from the risk-attitude test together with Thomas’ answer.

*Test item:*

Mr. J is an American captured by the enemy in World War II and placed in a prisoner-of-war camp. Conditions in the camp are quite bad, with long hours of hard physical labour and a barely sufficient diet. After spending several months in this camp, Mr. J notes the possibility of escape by concealing himself in a supply truck that shuttles in and out of the camp. Of course, there is no guarantee that the escape would prove successful. Recapture by the enemy could well mean execution.

What would you advise Mr. J. to do? To take the risk and attempt to escape or to avoid the risk and stay in the camp?

*Thomas' answer:*

I would go for the risky option and try to escape. By staying in the camp, he is risking starving to death anyway. Although the probability of a successful escape is not very high, I think that it is better to take the risk of being killed during the escape, rather than to continue suffering from starvation and hard physical labor in the camp without knowing whether and when it is going to end.

## Retraction of misinformation

You have completed several tasks related to the summary of a research study investigating the relationship between firefighters’ attitude to risk and successfulness in their job. The purpose of these tasks was to analyze peoples’ comprehension of scientific text and analytical thinking. For this purpose, the research summary presented to you had been invented. The mentioned study investigating the relationship between firefighters’ attitude to risk and successfulness in their job had never taken place. Please consider this information in the following tasks.

Confirm that you have carefully read the text above and are ready to continue by clicking on "I confirm".

🗹 I confirm

## Debriefing about the real purpose of the study

We would like to reveal the real aim of the survey. The survey was not designed to analyze peoples’ comprehension of scientific text and analytical thinking as we claimed at the beginning. This deception was necessary to divert your attention from **the real aim – the study of the belief perseverance bias and methods that might help to reduce the bias**.

# Appendix D: Measures of opinion

## Likert items

**Table 2: Nine pairs of oppositely worded Likert items tested in the preparatory study.** Subscripts P and N indicate a positive and negative formulation, respectively. The first eight pairs of Likert items were adopted as valid measures and used in the main study.

| 1. LIK_1P_ | 1. Risk-taking firefighters tend to be more successful in their job than risk-avoiding firefighters. |
| --- | --- |
| 1. LIK_1N_ | 1. Risk-avoiding firefighters tend to be more successful in their job than risk-taking firefighters. |
| 1. LIK_2P_ | 1. Successful firefighters tend to provide more risk-taking answers in risk-attitude tests than unsuccessful firefighters. |
| 1. LIK_2N_ | 1. Successful firefighters tend to provide more risk-avoiding answers in risk-attitude tests than unsuccessful firefighters. |
| 1. LIK_3P_ | 1. Emergency interventions conducted by risk-taking firefighters tend to result in less harm to health than emergency interventions conducted by risk-avoiding firefighters. |
| 1. LIK_3N_ | 1. Emergency interventions conducted by risk-avoiding firefighters tend to result in less harm to health than emergency interventions conducted by risk-taking firefighters. |
| 1. LIK_4P_ | 1. Successful firefighters tend to be more risk-taking than unsuccessful firefighters. |
| 1. LIK_4N_ | 1. Successful firefighters tend to be more risk-avoiding than unsuccessful firefighters. |
| 1. LIK_5P_ | 1. Firefighter teams with a prevailing number of risk-taking firefighters tend to be more successful in their interventions than firefighter teams with a prevailing number of risk-avoiding firefighters. |
| 1. LIK_5N_ | 1. Firefighter teams with a prevailing number of risk-avoiding firefighters tend to be more successful in their interventions than firefighter teams with a prevailing number of risk-taking firefighters. |
| 1. LIK_6P_ | 1. Should I become a firefighter, I would prefer to work in a team of risk-taking firefighters rather than in a team of risk-avoiding firefighters. |
| 1. LIK_6N_ | 1. Should I become a firefighter, I would prefer to work in a team of risk-avoiding firefighters rather than in a team of risk-taking firefighters. |
| 1. LIK_7P_ | 1. If I were a fire captain hiring firefighters into my new team, I would prefer to employ risk-taking firefighters rather than risk-avoiding firefighters. |
| 1. LIK_7N_ | 1. If I were a fire captain hiring firefighters into my new team, I would prefer to employ risk-avoiding firefighters rather than risk-taking firefighters. |
| 1. LIK_8P_ | 1. Emergency interventions conducted by risk-taking firefighters tend to be more successful than emergency interventions conducted by risk-avoiding firefighters. |
| 1. LIK_8N_ | 1. Emergency interventions conducted by risk-taking firefighters tend to be more successful than emergency interventions conducted by risk-avoiding firefighters. |
| 1. LIK_9P_ | 1. Emergency interventions conducted by risk-taking firefighters tend to result in fewer material damages than emergency interventions conducted by risk-avoiding firefighters. |
| 1. LIK_9N_ | 1. Emergency interventions conducted by risk-avoiding firefighters result in fewer material damages than emergency interventions conducted by risk-taking firefighters. |

## Phi-coefficient measures

**Table 3: Four phi-coefficient measures tested in the preparatory study.** All were adopted as valid measures and used in the main study.

| Phi_1_ | Consider the firefighters that show a risk-taking attitude on the job. What percentage of them are, in your opinion, successful in their job?  Consider the firefighters that show a risk-avoiding attitude on the job. What percentage of them are, in your opinion, successful in their job? |
| --- | --- |
| Phi_2_ (for the positive treatment) | Consider the firefighters that are successful in their job. What percentage of them have, in your opinion, a risk-taking attitude?  Consider the firefighters that are unsuccessful in their job. What percentage of them have, in your opinion risk-taking attitude? |
| Phi_2_ (for the negative treatment) | Consider the firefighters that are successful in their job. What percentage of them have, in your opinion, a risk-avoiding attitude?  Consider the firefighters that are unsuccessful in their job. What percentage of them have, in your opinion, a risk-avoiding attitude? |
| Phi_3_ (for the positive treatment) | Imagine that the study analyzing the relationship between risk attitude and successfulness was done once again, with 100 successful and 100 unsuccessful firefighters.  Estimate the number of successful firefighters that would show a risk-taking attitude in the risk-attitude test.  Estimate the number of unsuccessful firefighters that would show a risk-taking attitude in the risk-attitude test. |
| Phi_3_ (for the negative treatment) | Imagine that the study analyzing the relationship between risk attitude and successfulness was done once again, with 100 successful and 100 unsuccessful firefighters.  Estimate the number of successful firefighters that would show a risk-avoiding attitude in the risk-attitude test.  Estimate the number of unsuccessful firefighters that would show a risk-avoiding attitude in the risk-attitude test. |
| Phi_4_ | Imagine that the study analyzing the relationship between risk attitude and successfulness was done once again, with 100 risk-taking firefighters and 100 risk-avoiding firefighters.  Estimate how many of the risk-taking firefighters would be successful in their job. |

# Appendix E: Debiasing techniques

## Counter-explanation

You already know that the research study suggesting that the more risk-taking attitude firefighters have, the more successful they are in their job had been invented. Thus, **it is possible that the opposite may be true**, i.e. the more risk-avoiding attitude the firefighters have, the more successful they are in their job. **Now try to think of and write down at least three arguments why this might be true**.

Example of an argument: Risk-avoiding firefighters are more successful than risk-taking firefighters because they do not rush headlong into danger without thinking things through first.

## Counter-speech

You already know that the research study suggesting that the more risk-taking attitude firefighters have, the more successful they are in their job had been invented. **Thus, it is possible that the opposite may be true**, i.e. the more risk-avoiding attitude the firefighters have, the more successful they are in their job. There are actually several arguments supporting this hypothesis**.** For example:

1. Risk-avoiding firefighters, unlike risk-taking firefighters, **are likely not to rush** **headlong into danger** without thinking things through first. This may lead to the higher successfulness of their interventions.
2. Risk-avoiding firefighters **probably get injured less often** than risk-avoiding firefighters during their interventions. This is another factor that may lead to more success.
3. Risk-avoiding firefighters seem not to do their job because they would like to become heroes but because **they want to help others.** This suggests that they **are more responsible** and herewith more successful than risk-taking firefighters.

Spend some time thinking about these arguments and then try to think of other possible arguments supporting this hypothesis.

## Awareness training

You already know that the research study suggesting that the more risk-taking attitude firefighters have, the more successful they are in their job had been invented. Therefore, the information in the research study should theoretically have no influence on your opinion regarding the potential relationship between the willingness to take or avoid risk and successfulness as a firefighter. Nevertheless, the opposite is likely to be true. It appears that people often insist on opinions or hypotheses, even when they know that they are based on wrong information. This irrational behavior is called ‘*belief perseverance*’.

The following situation can further illustrate the effect of belief perseverance: Emily tells Oliver that the new fellow student Paul is not particularly clever and advises him against working in a group with Paul. After a few days, Oliver finds out that there was a misunderstanding and that Emily did not speak about Paul but Tim. If Oliver were to act rationally, he would now be completely free of prejudices against Paul since his original opinion about Paul turned out to be based on wrong information. Nevertheless, due to belief perseverance, Oliver continues to avoid working with Paul, even though he knows Emily has talked about someone else.

It is important that we create and change our opinion and make decisions freely without being manipulated by misinformation. Thus, we should be aware of this trap in the form of ‘belief perseverance’ whenever we encounter misinformation.
